# Rotenone ameliorates chronic renal injury caused by acute ischemia/reperfusion

**DOI:** 10.18632/oncotarget.24733

**Published:** 2018-05-11

**Authors:** Wen Zhang, Yugen Sha, Ke Wei, Chunfeng Wu, Dan Ding, Yunwen Yang, Chunhua Zhu, Yue Zhang, Guixia Ding, Aihua Zhang, Zhanjun Jia, Songming Huang

**Affiliations:** ^1^ Department of Nephrology, Children's Hospital of Nanjing Medical University, Nanjing 210008, China; ^2^ Jiangsu Key Laboratory of Pediatrics, Nanjing 210029, China; ^3^ Nanjing Key Laboratory of Pediatrics, Nanjing 210008, China

**Keywords:** rotenone, mitochondria, AKI, CKD

## Abstract

Acute kidney injury (AKI) has been widely recognized as an important risk factor leading to the occurrence and progression of chronic kidney disease (CKD). Thus, development of the strategies in retarding the transition of AKI to CKD is becoming a hot research field. Recently, accumulating evidence suggested a pathogenic role of mitochondrial dysfunction in both AKI and CKD. Therefore, in the present study, we evaluated the effect of mitochondrial complex 1 inhibition by rotenone on the chronic renal damage induced by acute ischemia-reperfusion. The mice were treated with 45 min unilateral renal ischemia and reperfusion (I/R) to induce an acute renal injury. After three days of I/R injury, rotenone at a dose of 200 ppm in food was administered to the mice. Strikingly, after three weeks treatment with rotenone, we found that the unilateral I/R-induced tubular damage, tubulointerstitial fibrosis were all attenuated by rotenone as determined by the tubular injury score, Masson staining, and the levels of collagen-I, collagen-III, fibronectin, PAI-1, and TGF-β. Meanwhile, the enhanced inflammatory markers of TNF-α, IL-1β, IL-6, and IL-18 and apoptotic markers of Bax and caspase-3 were all significantly blunted by inhibiting mitochondrial complex-1. Moreover, rotenone treatment also partially protected the mitochondria as shown by the restoration of mitochondrial SOD (SOD2), ATPB, and mitochondrial DNA copy number. These findings suggested that inhibition of mitochondrial complex-1 activity by rotenone could retard the progression of AKI to CKD probably via protecting the mitochondrial function to some extent.

## INTRODUCTION

Acute kidney injury (AKI) has been estimated with a mortality rate of ~50% [1]. To date, no effective treatments are available to improve such a status. Furthermore, AKI also serves as an important risk factor for the development of CKD [2]. After an episode of AKI, some patients developed progressive and persistent deterioration of renal function [3]. Moreover, these patients were more likely to progress into the end-stage renal disease (ESRD) compared to patients without a history of AKI [2]. Thus, more and more researches focused on the understanding of the mechanisms underlying the transition of AKI to CKD and the identification of possible therapeutic targets [4].

Some researches revealed an abnormal change of mitochondria in AKI such as ischemia-reperfusion [5] and CKD models of UUO [6–8] and 5/6 nephrectomy [9]. Mitochondrial damage enhanced reactive oxygen species (ROS) production and increased inflammation [10, 11]. In addition, the dysfunction of mitochondria also causes ATP depletion and the release of proapoptotic factors like cytochrome C and mitochondrial DNA, which could result in the cell injury via oxidative damage of DNA and protein, apoptotic response, and subsequent inflammation and fibrosis [12, 13]. The agents that could inhibit the over-activation of mitochondrial and improve the mitochondrial function provide a promising way in the treatment of kidney diseases.

Unilateral renal ischemia-reperfusion model was established to study the mechanisms and therapies of AKI to CKD in mice [14]. Our previous study demonstrated that treatment with a mitochondrial complex-1 inhibitor rotenone in UUO model could improve the mitochondrial dysfunction and attenuate the kidney damage [15], suggesting an important role of mitochondrial dysfunction in the pathogenesis of obstructive chronic kidney disease. However, whether mitochondrial dysfunction plays a role in the transition of AKI to CKD needs to be defined. Therefore, in the present study, employing a unilateral renal I/R-induced AKI to CKD model and the mitochondrial complex-1 inhibitor rotenone, we investigated the role of mitochondrial complex-1 inhibitor rotenone in retarding the progression of AKI to CKD.

## RESULTS

### Rotenone treatment improved renal tubular injury after unilateral renal ischemia/reperfusion

**Figure 1:**
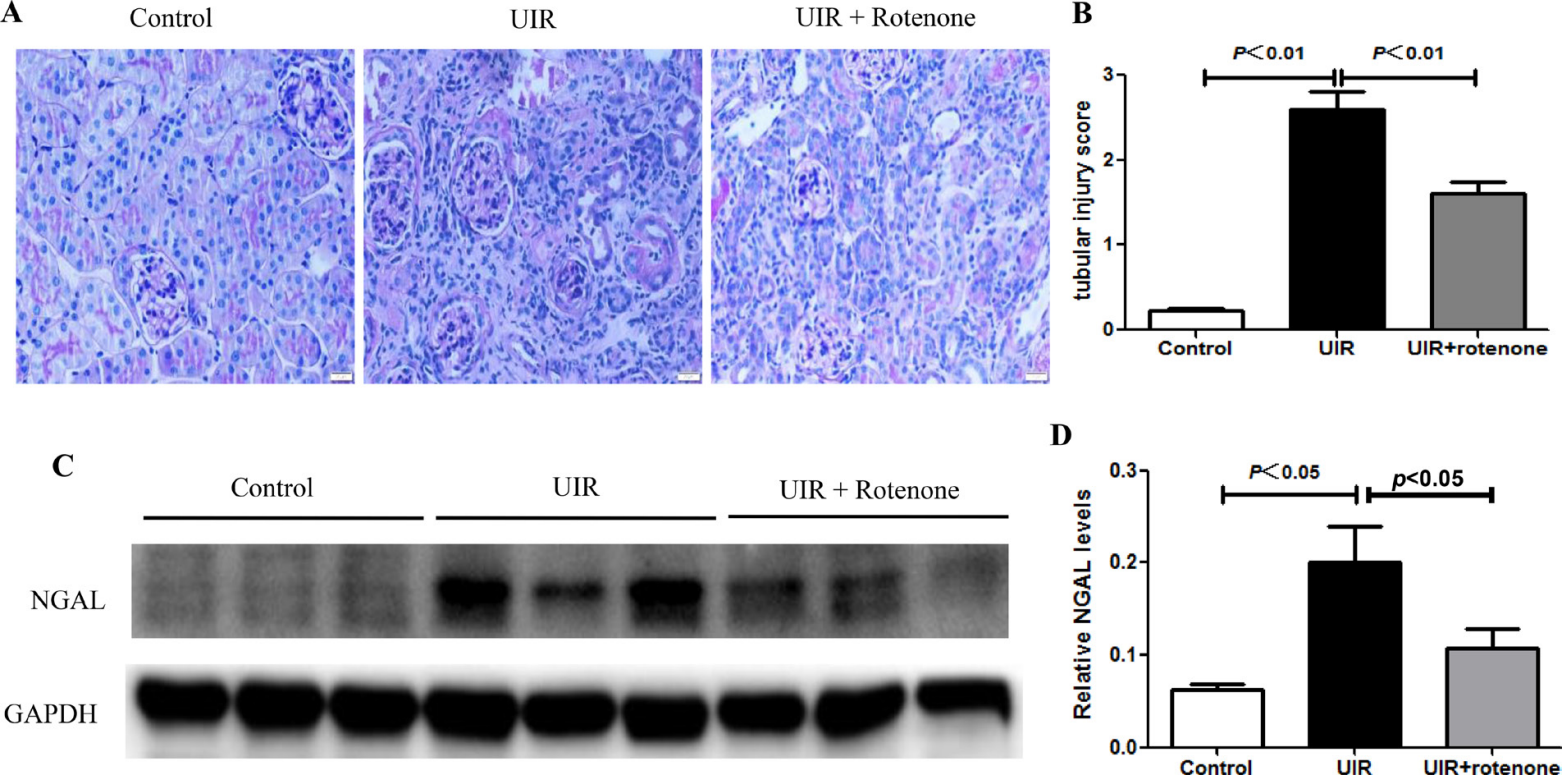
Rotenone treatment improved renal tubular injury in unilateral I/R mice (**A**) Representative images of renal PAS staining in control and uniliteral I/R mice with or without rotenone treatment. (**B**) Tubular injury score. (**C**) Western blot of NGAL. GAPDH was used as loading control. (**D**) Densitometry of Western blot in A. Data were presented as means ± SE, *n* = 6 in each group.

At first, we performed PAS staining to observe the tubular structure damage after 3 weeks rotenone treatment in unilateral renal ischemia/reperfusion mice. As shown by the data, the loss of brush border, epithelial cell atrophy and flattening, and tubular lumen dilation were all attenuated by rotenone treatment (Figure 1A and 1B). NGAL is a kwon tubular injury marker in AKI. Recently, NGAL was also found to be upregulated in chronic kidney injury. In the present study, we observed the regulation of NGAL in I/R kidney (Figure 1C and 1D). Significantly, rotenone treatment suppressed the upregulation of NGAL protein in I/R kidneys (Figure 1C and 1D). These results demonstrated a beneficial effect of rotenone treatment on protecting the renal tubules during the transition of AKI to CKD.

### Rotenone treatment attenuated tubulointerstitial fibrosis after unilateral renal ischemia/reperfusion

**Figure 2:**
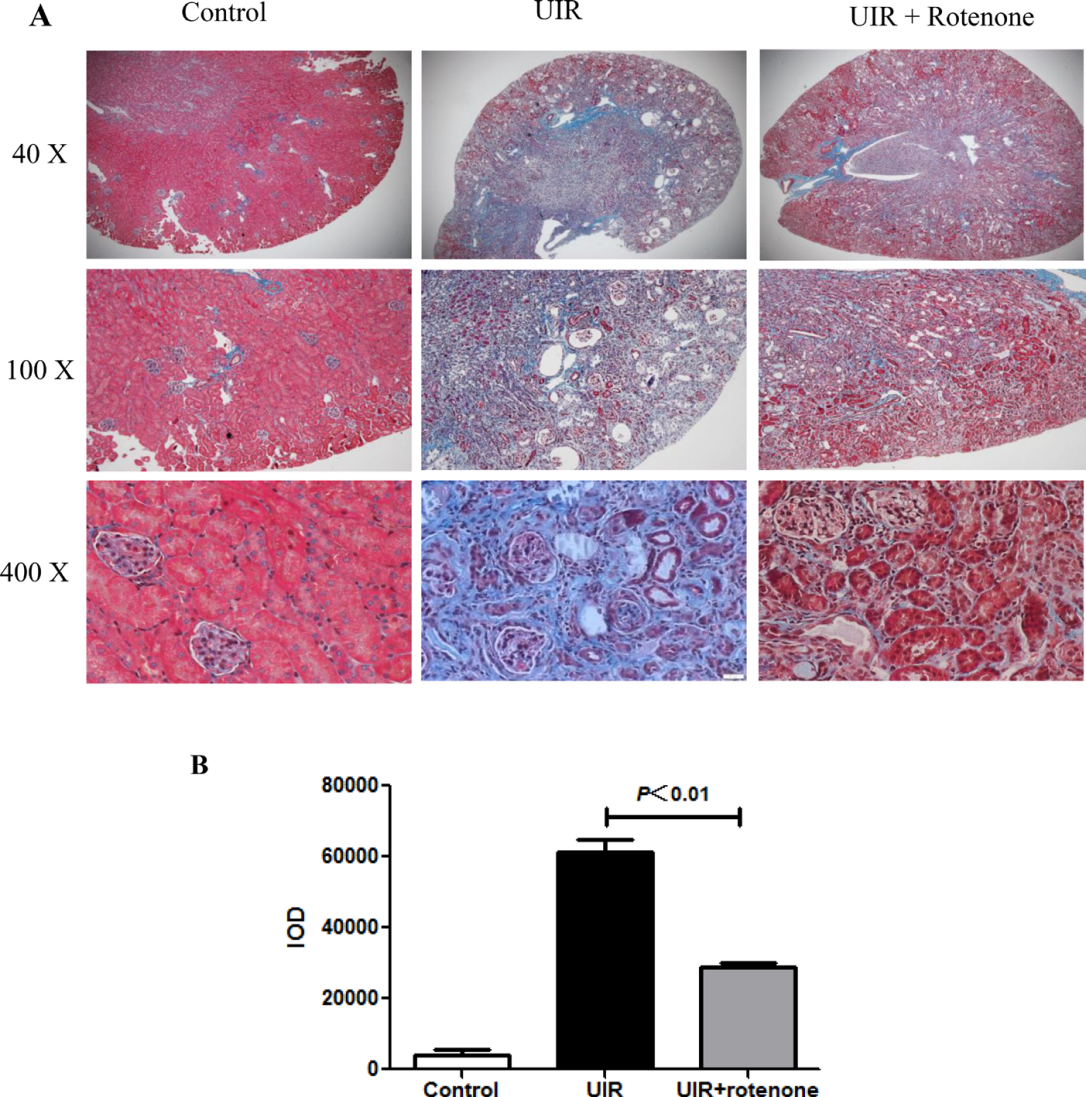
Rotenone treatment attenuated the deposition of extracellular matrixin the kidneys of uniliteral I/R mice After 3 days of unilateral I/R, mice were treated with or without rotenone (200 ppm) for 3 weeks. (**A**) Representative images of Masson staining. (**B**) Quantification of extracellular matrix deposition (blue staining) in kidneys. Data were presented as Means ± SE, *n* = 6 in each group.

**Figure 3:**
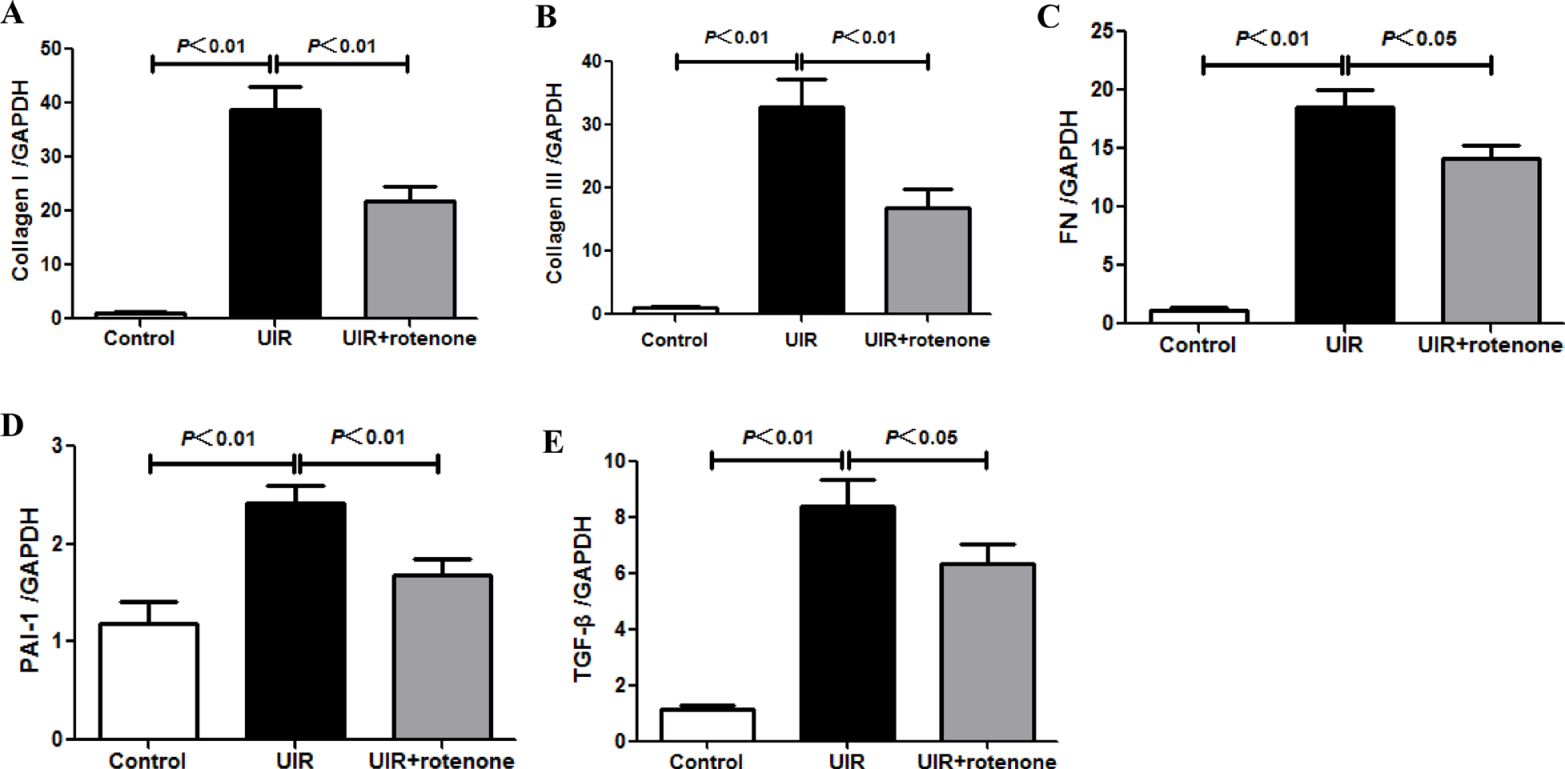
Rotenone treatment blunted the upregulation of fibrotic markers in unilateral I/R kidneys (**A–E**) qRT-PCR analyses of collagen I (A), collagen III (B), FN (C), PAI-1 (D), and TGF-β (E) mRNA levels. Data were presented as means ± SE, *n* = 6 in each group.

**Figure 4:**
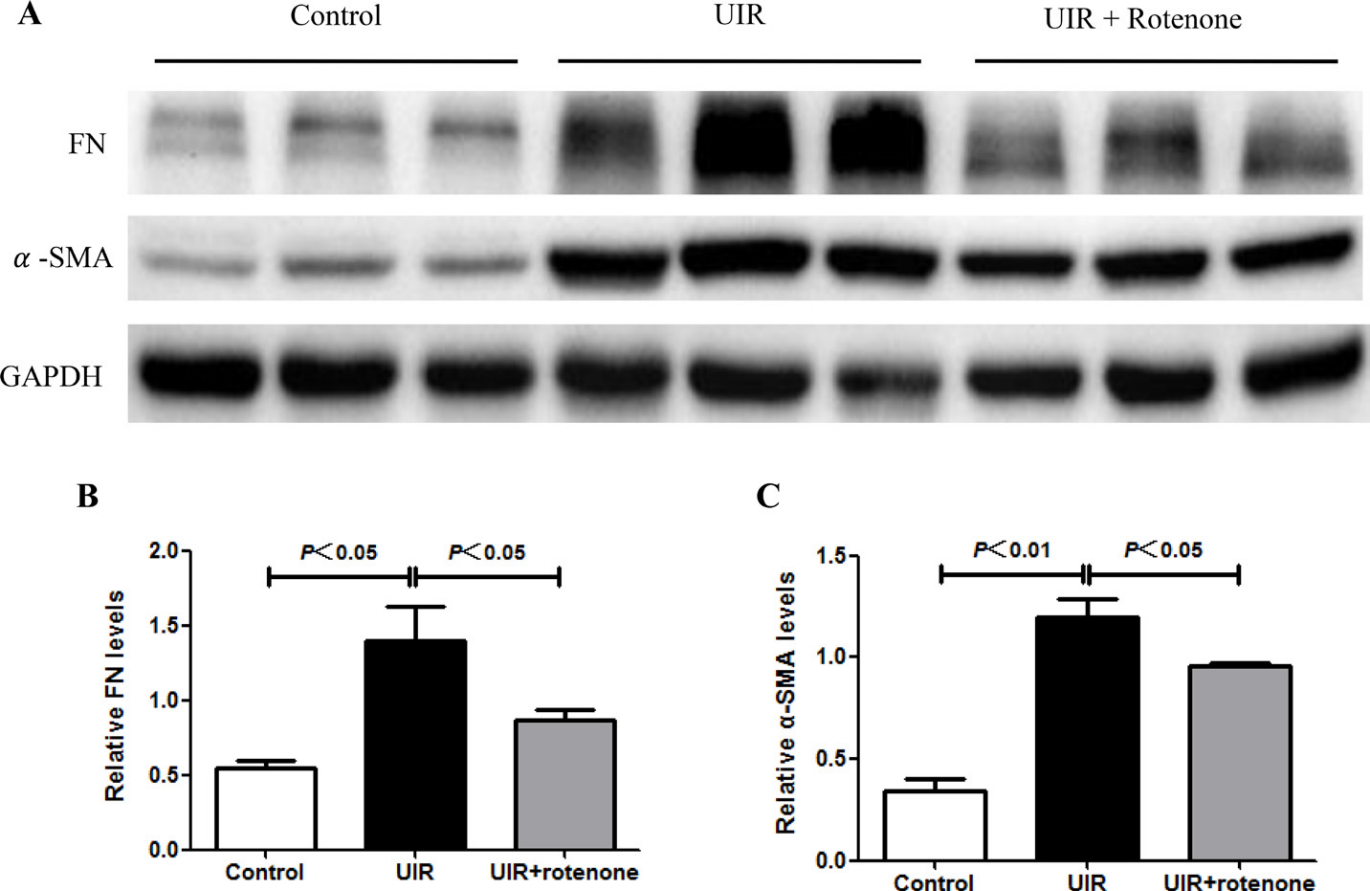
Rotenone treatment blocked the upregulation of fibronectin and α-SMA in unilateral I/R kidneys (**A**) Fibrotic markers of FN and α-SMA were determined by Western blotting. GAPDH was used as loading control. (**B**) Densitometry of Western blots in A. (**C**) Data were presented as means ± SE, *n* = 6 in each group.

Next, we evaluated the fibrotic status in I/R kidneys with or without 3 weeks rotenone treatment. By Masson staining, 24 days unilateral I/R caused a robust tubulointerstitial matrix deposition in line with a striking kidney atrophy (Figure 2A and 2B). After 3 weeks rotenone treatment, the fibrotic response was significantly improved (Figure 2A and 2B). At the same time, we performed qRT-PCR to test the mRNA levels of collagen I, collagen III, FN, PAI-1, and TGF-β. As shown in Figure 3, all these parameters were markedly upregulated in I/R kidneys, which was partially but significantly blunted after 3 weeks rotenone treatment (Figure 3A–3E). By Western blotting, we further confirmed that the upregulation of FN and α-SMA in I/R kidneys was also blunted by rotenone treatment (Figure 4A–4C). These data demonstrated that inhibition of mitochondrial complex-1 activity retarded the chronic renal damage during the transition of AKI to CKD.

### Rotenone treatment ameliorated inflammatory and apoptotic responses after unilateral renal ischemia/reperfusion

**Figure 5:**
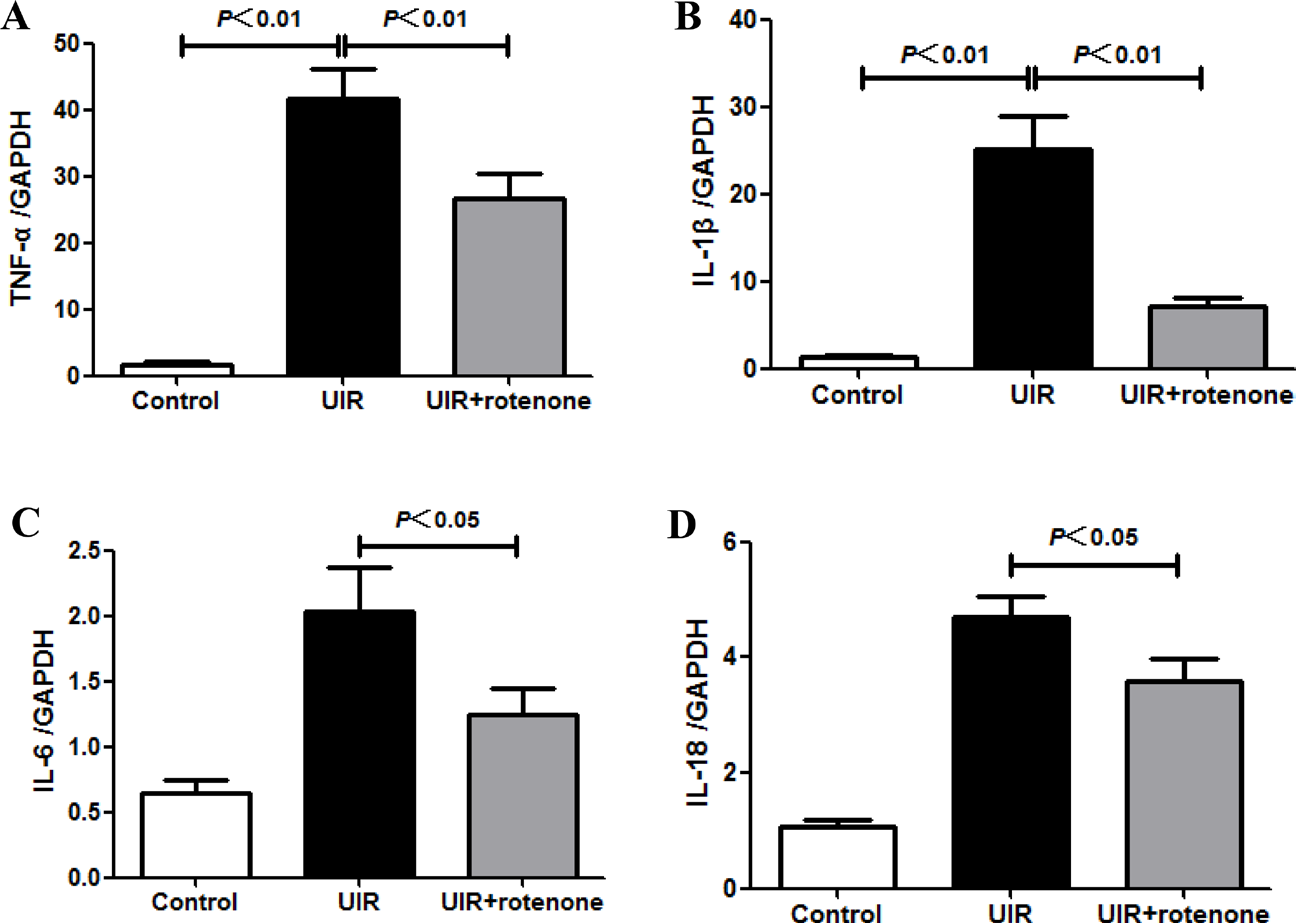
Rotenone treatment blunted inflammatory response in unilateral I/R kidneys (**A–D**) qRT-PCR analyses of TNF-α (A), IL-1β (B), IL-6 (C), and IL-18 (D). Data were presented as means ± SE, *n* = 6 in each group.

**Figure 6:**
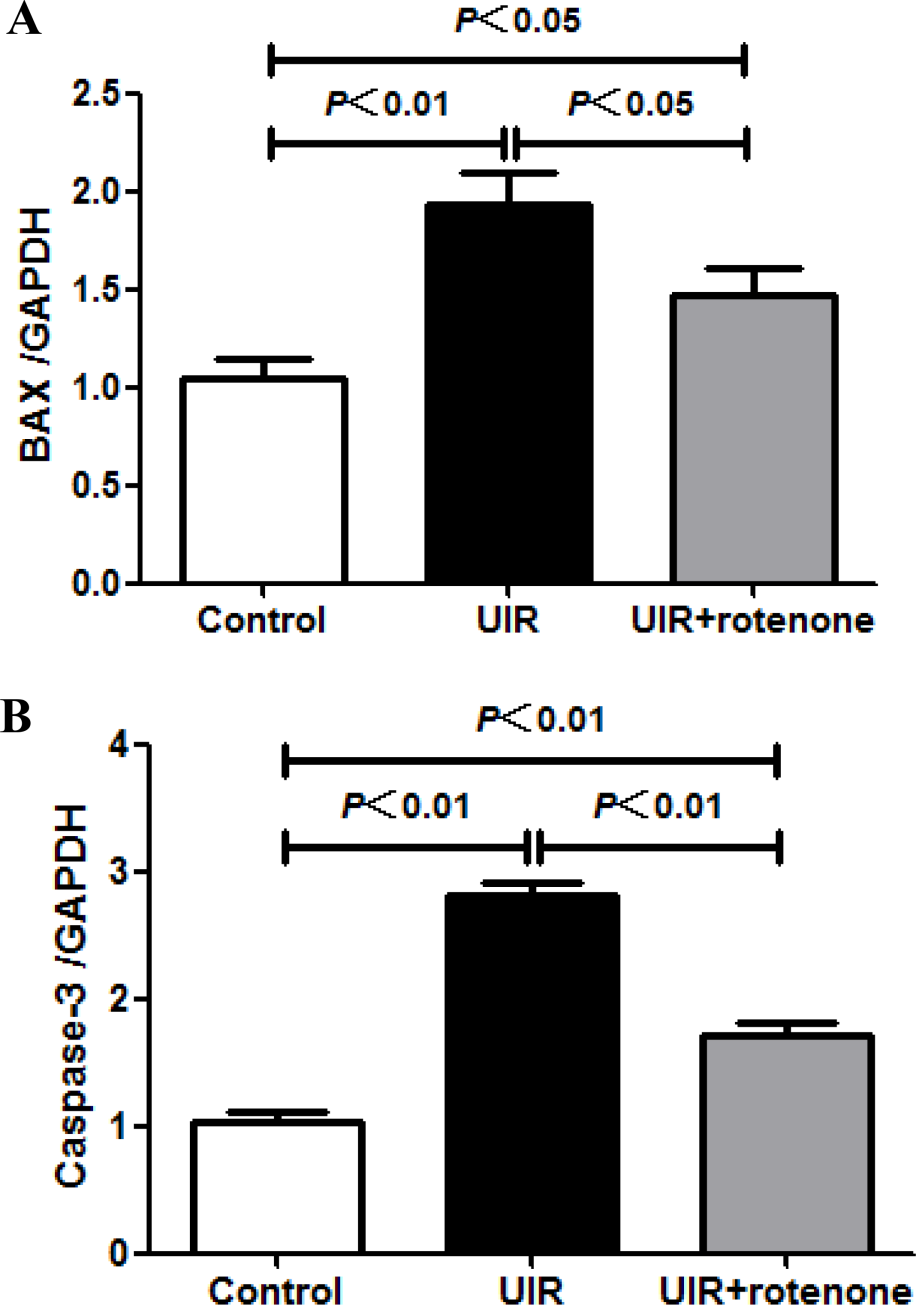
Effect of rotenone treatment on the expressions of Bax and caspase-3 in unilateral I/R kidneys (**A**) qRT-PCR analysis of Bax. (**B**) qRT-PCR analysis of caspase-3. Data were presented as means ± SE, *n* = 6 in each group.

By PAS staining, we found augmented infiltrating cells in I/R kidneys, which was ameliorated in rotenone-treated animals (Figure 1A). Furthermore, we observed that the enhanced inflammatory markers of TNF-β, IL-1β, IL-6, and IL-18 in I/R kidneys were significantly blocked by rotenone administration (Figure 5A–5D). At the same time, the upregulation of apoptosis-related genes of Bax and caspase-3 was also significantly downregulated in the kidneys of rotenone-treated animals (Figure 6A and 6B). These results suggested anti-inflammatory and anti-apoptotic effects of rotenone during the transition of AKI to CKD.

### Rotenone treatment attenuated mitochondria damage after unilateral renal ischemia/reperfusion

**Figure 7:**
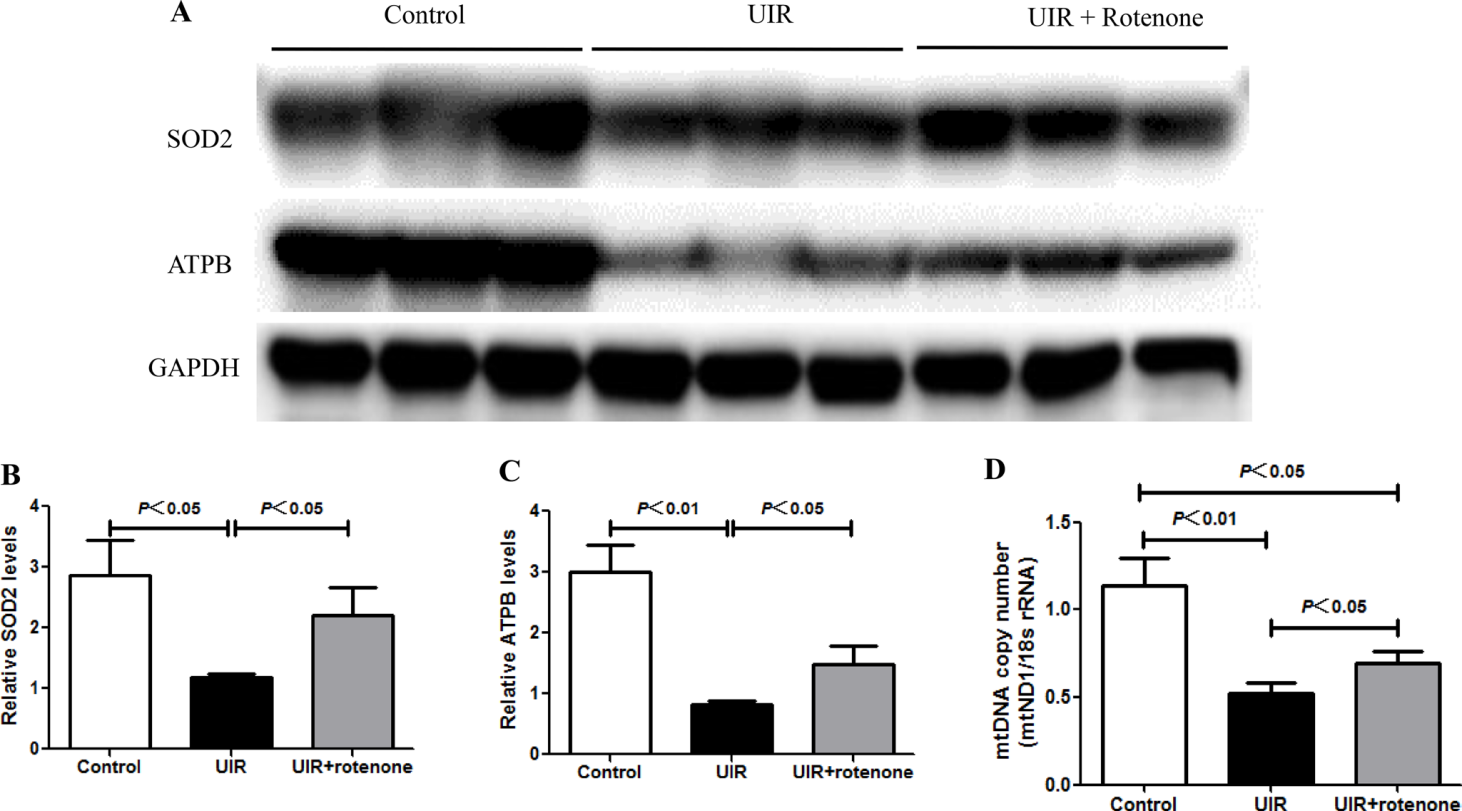
Rotenone treatment improved the mitochondrial abnormality in unilateral I/R kidneys (**A**) Western blotting analyses of SOD2 and ATPB. GAPDH was used as loading control. (**B** and **C**) Densitometry of Western blots in A. (**D**) mtDNA copy number was determined by qRT-PCR. Data were presented as means ± SE, *n* = 6 in each group.

Mitochondrial dysfunction has been recognized as one of pathogenic factors in both AKI and CKD. Thus, we evaluated the mitochondrial status by the examination of mitochondrial DNA copy number, and the protein levels of mitochondrial SOD (SOD2) and ATPB. As shown by the data (Figure 7A–7D), the reduction of mitochondrial SOD, mitochondrial ATPB, and mitochondrial DNA copy number was significantly restored by rotenone treatment in I/R kidneys. These data suggested a protective effect of rotenone treatment on mitochondria.

### Safety evaluation of rotenone treatment in mice

**Figure 8:**
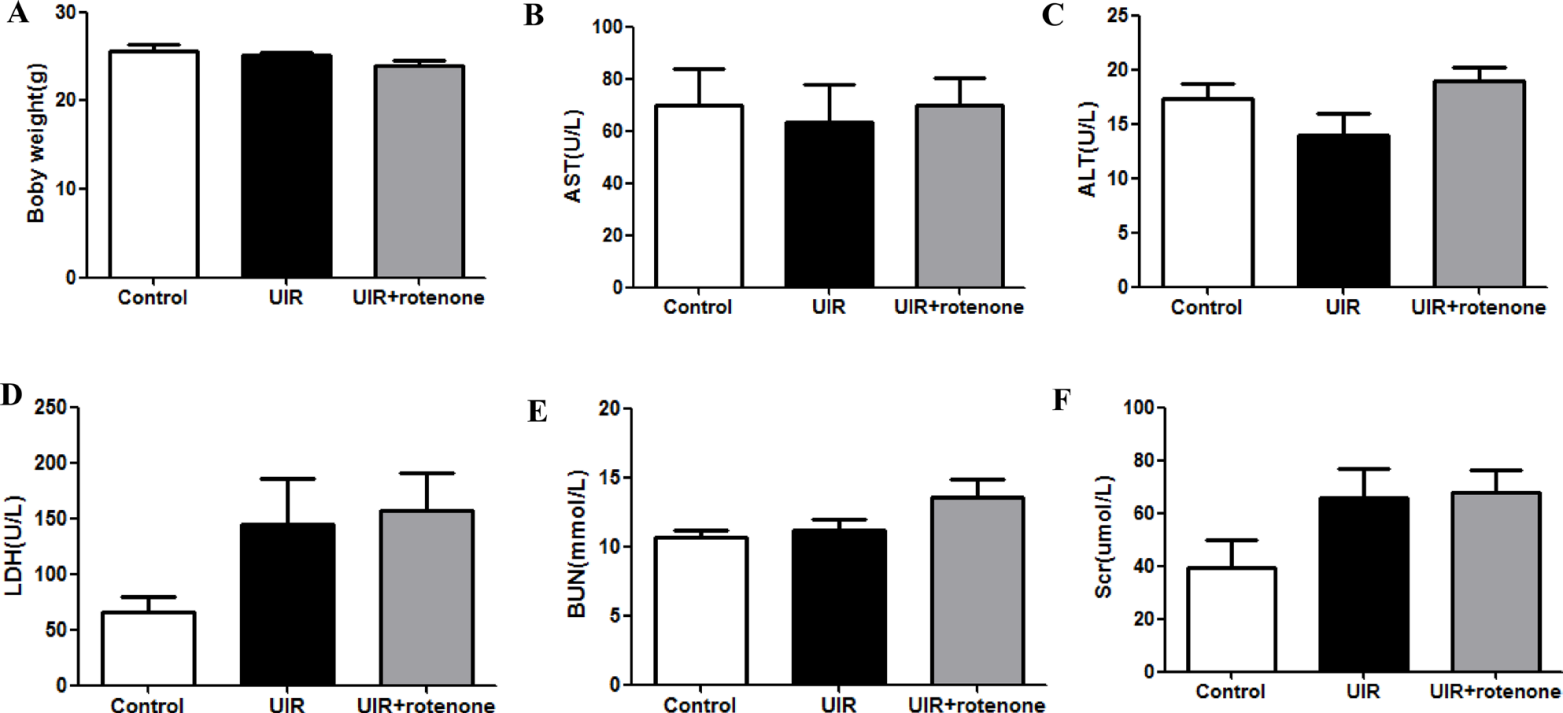
Safety evaluation of rotenone treatment in mice (**A**) Body weight after 3-week rotenone treatment. (**B–F**) blood levels of AST (B), ALT (C), LDH (D), BUN (E), and Scr (F). Data were presented as means ± SE, *n* = 6 in each group.

It is known that rotenone at a higher dose was used to induce mitochondrial dysfunction and disease models [16, 17]. In the present study, we chose a relatively lower dose of rotenone (200 ppm) to block the activity of abnormal mitochondria during a pathological condition, which might attenuate the mitochondrial dysfunction-associated tissue damage. Thus, we examined the body weight and the blood levels of AST, ALT, LDH, BUN, and Creatinine to evaluate the safety of 3 weeks rotenone treatment at a dose of 200 ppm in food. As shown by the data, compared with vehicle-treated unilateral I/R mice, rotenone treatment for 3 weeks did not significantly affect above parameters (Figure 8A–8F) in line with a similar food intake between groups (unilateral I/R mice vs. unilateral I/R + Rotenone mice: 5.2 ± 0.2 vs. 4.9 ± 0.3 g/day, *p >* 0.05), indicating that 200 ppm rotenone treatment did not affect the functions of liver, heart, and kidney. Thus, three weeks treatment of rotenone at 200 ppm in food was largely safe for the mice.

## DISCUSSION

Acute kidney injury could be induced by drugs, toxins, kidney ischemia, and sepsis [18, 19]. Recent clinical studies demonstrated that AKI served as an independent risk factor for the development of CKD and ESRD [20]. Experimental studies on mechanisms underlying the progression from AKI to CKD indicated a persistent inflammatory response [21], mitochondrial injury, and renal microvasculature alteration [22, 23], which may finally contribute to the development of CKD and ESRD. In the present study, we established a unilateral ischemia/reperfusion (45 min) mouse model without contralateral nephrectomy. Forty-five min ischemia could induce a relatively more severe I/R injury, which subsequently resulted in the typical chronic kidney damage. This model can avoid the high mortality shown in bilateral renal I/R because the contralateral kidney is normal. Overall, this is a satisfactory model of AKI to CKD.

Ischemia causes mitochondrial damage in renal cells [24, 25]. Mitochondrial damage results in the reduction of ATP, the increment of ROS, and the release of proapoptotic factors such as cytochrome C and mitochondrial DNA, which subsequently causes cellular and tissue injury [26, 27]. However, it has not been known whether mitochondrial dysfunction plays a role during AKI to CKD progression. We suppose that the mitochondrial dysfunction in kidney is still existed after AKI, and a suitable control of these dysfunctional mitochondria might be helpful in retarding the transition of AKI to CKD. In agreement with this concept, we previously reported that a mitochondrial complex-1 inhibitor rotenone could ameliorate mitochondrial dysfunction and attenuate renal fibrosis in a UUO CKD model [15]. In present study, after 3 days of ischemia/reperfusion, rotenone treatment at a dose of 200 ppm in food for 3 weeks significantly improved the renal fibrosis, inflammation and mitochondrial abnormality.

Rotenone is a known inhibitor of mitochondrial complex-1. High dose of rotenone was widely used to induced mitochondrial disease models [16, 17]. However, a number of studies also indicated that a lower dose of rotenone could be protective in many diseases without obvious detrimental effect on animals, organs, and cells [15]. Thus, the evidence highly suggested a potential of a suitable mitochondrial inhibition in treating acute and chronic diseases including kidney diseases possibly through inhibiting the aberrant activity of the injured mitochondria.

In summary, the present study demonstrated that after AKI caused by 45 min ischemia and reperfusion, post administration (during the recovery stage) with rotenone protected the renal tubules, blocked the renal fibrosis, attenuated the inflammation and apoptotic responses, and improved mitochondrial abnormality. These findings not only suggested a role of mitochondrial dysfunction in the pathogenesis of AKI to CKD transition but also indicated a potential of a suitable mitochondrial inhibition in the therapies of AKI to CKD.

## MATERIALS AND METHODS

### Reagents and antibodies

Rotenone was purchased from Sigma (St. Louis, MO). Antibodies against fibronectin, smooth muscle actin (α-SMA), ATPB, SOD2, and GAPDH were purchased from Proteintech (Wuhan, China), Antibody against NGAL was from Abcam (Cambridge, MA).

### Animals

C57BL/6Jmice were originally obtained from Jackson lab. This mouse colony was propagated at the Nanjing Medical University. For all experiments, 8–12 weeks old male mice were used. All mice were maintained on a 12 h light-dark cycle in a temperature-controlled (22 ± 2°C) room, were fed a standard rodent diet, and were allowed to free access to drinking water. The procedures were approved by Nanjing Medical University Institutional Animal Care and Use Committees.

### Unilateral renal ischemia/reperfusion injury experimental protocol and rotenone treatment

Mice were anesthetized with 2.0% isoflurane and unilateral renal ischemia was induced by application of microvascular clamps around left renal pedicles for 45 minutes, without contralateral nephrectomy. The abdominal incision was closed with a 4–0 suture. Three days (72 h) after this procedure, the gelled diet with or without rotenone at a dose of 200 ppm was given to the unilateral renal I/R mice. The sham control mice were treated with gelled diet without rotenone. Before rotenone treatment, the animals were fed control gelled diet (no rotenone). The gelled diet with rotenone was made by melting agar (1% by weight) in water (50%), cooling (52°C), and adding the rotenone, ground chow (49%). Rotenone was absent in the control gelled diet. After three-week treatment of rotenone, mice (*N* = 6 per group) were sacrificed and the kidney tissues were harvested for the evaluation of gene and protein expressions and histology.

### Renal histology

Harvested kidney tissues from mice were fixed with 4% paraformaldehyde, embedded in paraffin, and sectioned transversely. Paraffin sections (3 μm) were stained with periodic acid–Schiff and examined by light microscopy. Masson trichrome staining was used to assess tubulointerstitial fibrosis.

### Tubular injury score

Kidney tissues were fixed by direct immersion in 4% paraformaldehyde. After embedding in paraffin, 3 mm sections were prepared and stained with periodic acid Schiff (PAS) and analyzed with light microscope. The percentage of tubular injury parameters containing epithelial flattening, tubular atrophy, tubular dilatation, and brush border loss were estimated by a pathologist who was blind to the identity of the specimen using a 4-point scale in ten randomly chosen, nonoverlapping fields (200× magnification). Degree of injury was graded on a scale from 0 to 4: 0 = normal; 1 = mild, involvement of less than 25% of the cortex; 2 = moderate, involvement of 25 to 50% of the cortex; 3 = severe, involvement of 50 to 75% of the cortex; and 4 = extensive damage involving > 75% of the cortex.

### Quantitative real-time-PCR (qRT-PCR)

Total DNA and RNA from kidney cortex tissues were isolated using the DNeasy Tissue Kit (Qiagen, Valencia, CA) and TRIzol reagent (TaKaRa), respectively. Oligonucleotides were designed using Primer5 software (available at http://frodo.wi.mit.edu/) and synthesized by Invitrogen. Reverse transcription was performed using the Promega Reverse Transcription System according to the manufacturer's protocol (Madison, WI). Real-time PCR amplification was performed using the ABI 7500 Real-Time PCR Detection System (Foster City, CA) by using SYBR Premix Ex Taq (TaKaRa). The cycling program consisted of a preliminary denaturation (95°C for 10 min), followed by 40 cycles (95°C for 15 s and 60°C for 1 min). The relative mitochondrial DNA copy number was normalized to the 18S rRNA level encoded by the nuclear DNA, and mRNA levels were normalized to GAPDH and calculated using the comparative cycle threshold (ΔΔCt) method. The primer sequences were shown in Table 1.

**Table 1:** Sequences of primers for qRT-PCR

GENE	PRIMER SEQUENCE
GAPDH	5′-GTCTTCACTACCATGGAGAAGG-3′ 5′-TCATGGATGACCTTGGCCAG-3′
TNF-α	5′-TCCCCAAAGGGATGAGAAG-3′ 5′-CACTTGGTGGTTTGCTACGA-3′
IL-6	5′-GCTTAGGCATAACGCACT-3′ 5′-GGAAATCGTGGAAATGAG-3′
IL-18	5′-GACTCTTGCGTCAACTTCAAGG -3′ 5′-CAGGCTGTCTTTTGTCAACGA -3′
IL-1β	5′-ACTGTGAAATGCCACCTTTTG-3′ 5′-TGTTGATGTGCTGCTGTGAG-3′
PAI-1	5′-CACGCTACTTCCTCCTCAAG-3′ 5′-CTCTGTCTTCATCAGCTGGC-3′
FN	5′-CGTGGAGCAAGAAGGACAA-3′ 5′-GTGAGTCTGCGGTTGGTAAA-3′
Collagen I	5′-CCGGCTCCTGCTCCTCTT-3′ 5′-TTGCACGTCATCGCACAC-3′
Collagen III	5′-TGGTTTCTTCTCACCCTTCTT-3′ 5′-CAAATGGGATCTCTGGGTTG-3′
TGF-β1	5′-TACGCCTGAGTGGCTGTCTT-3′ 5′-CGTGGAGTTTGTTATCTTTGCT-3′
mtND1	5′-AATCGCCATAGCCTTCCTAACAT-3′ 5′-GGCGTCTGCAAATGGTTGTAA-3′
18s rRNA	5′-TTCGGAACTGAGGCCATGATT-3′ 5′-TTTCGCTCTGGTCCGTCTTG-3′
Bax	5′-AGACAGGGGCCTTTTTGCTAC-3′ 5′-AATTCGCCGGAGACACTCG-3′
Caspase-3	5′-ATGGGAGCAAGTCAGTGGA-3′ 5′-GGCTTAGAATCACACACACAAAG-3′

### Western blotting

kidney cortex tissues were lysed using a protein lysis buffer containing 50 mM Tris, 150 mM NaCl, 10 Mm EDTA, 1% Triton X-100, 200 mM sodium fluoride, and 4 mM sodium orthovanadate as a protease inhibitor (pH 7.5). Immunoblotting was then performed using primary antibodies against fibronectin (1:500), α-SMA (1:500), ATPB (1:1000), SOD2 (1:1000), NGAL (1:1000), and GAPDH (1:1000), followed by addition of HRP-labeled secondary antibodies. The blots were visualized using the Amersham Enhanced Chemiluminescence detection system (Amersham, Little Chalfont, Buckinghamshire, UK). Band intensity was measured using Image J software (NIH, Bethesda, MD, USA).

### Statistical analysis

All of the data were presented as means ± SE. The statistical analysis was performed using ANOVA followed by Bonferroni's test or unpaired Student's *t*-test with SPSS 16 statistical software. *P <* 0.05 was considered significant.

## Abbreviations

AKI: acute kidney injury
CKD: chronic kidney disease
I/R: ischemia and reperfusion
UIR: unilateral renal ischemia and reperfusion
PAI-1: plasminogen activator inhibitor-1
TGF-β: transforming growth factor-β
TNF-α: tumor necrosis factor-α
IL: interleukin
SOD: superoxide dismutase
CYTB: cytochrome b
ESRD: end-stage renal disease
UUO: unilateral ureteral obstruction
ROS: reactive oxygen species
ATP: adenosine-5’-triphosphate
PAS: periodic acid–Schiff
mtDNA: mitochondrial DNA
mtND1: mitochondrial NADH dehydrogenase subunit-1
FN: fibronectin
α-SMA: smooth muscle actin
ATPB: a subunit B of mitochondrial ATP synthase
AST: aspartate aminotransferase
ALT: alanine transaminase
LDH: lactate dehydrogenase
BUN: blood urea nitrogen
Scr: serum creatinine
GAPDH: glyceraldehyde-3-phosphate dehydrogenase
